# Supplementary material for: A glycine-rich PE_PGRS protein governs mycobacterial actin-based motility
Source: Nat Commun. 2022 Jun 24;13:3608. doi: 10.1038/s41467-022-31333-0 (PMC9232537; doi:10.1038/s41467-022-31333-0)
Supplement: Supplementary file 1 — Supplementary Information [file 41467_2022_31333_MOESM1_ESM.pdf]

Supplementary Information:

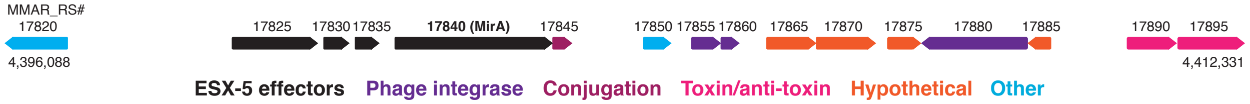
MMAR_RS#
17820
4,396,088
17825
17830
17835
17840 (MirA)
17845
17850
17855
17860
17865
17870
17875
17880
17885
17890
17895
4,412,331
ESX-5 effectors
Phage integrase
Conjugation
Toxin/anti-toxin
Hypothetical
Other

Supplementary Fig. 1. *mirA* locus is adjacent to factors associated with horizontal gene transfer. The *mirA* genetic locus (MMAR_RS17825 to MMAR_RS17840) is adjacent to elements associated with horizontal gene transfer, including genes coding for functions involved in conjugation, mycobacteriophage phage transduction, or a toxin/antitoxin system. Additionally, the locus is flanked by an abnormal level of intergenic DNA. ~124 nt is the average intergenic DNA in the *M. marinum* genome whereas the MirA locus is flanked by 2,173 nt on the 5’ end and 991 nt on the 3’ end.

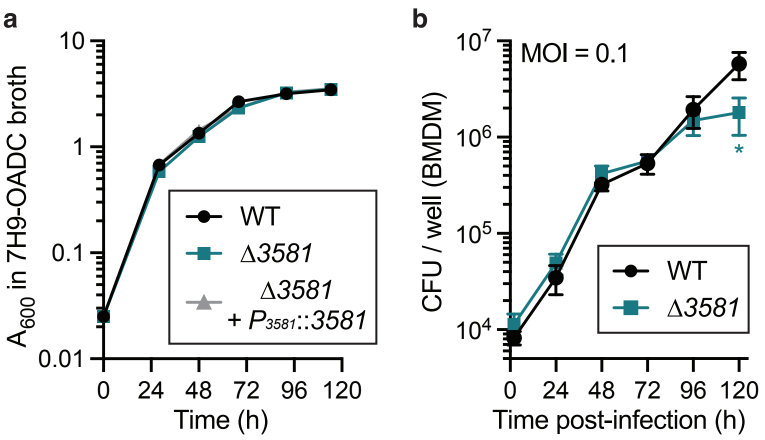
a
10
1
0.1
0.01
A600 in 7H9-OADC broth
0
24
48
72
96
120
Time (h)
WT
Δ3581
Δ3581
+ P3581::3581
b
MOI = 0.1
10^7
10^6
10^5
10^4
CFU / well (BMDM)
*
WT
Δ3581
0
24
48
72
96
120
Time post-infection (h)

Supplementary Fig. 2. The Δ*MMAR_3581* mutant exhibits normal growth kinetics. a Growth curve of wild type, Δ*3581*, and Δ*3581* + P_{3581}-*3581 M. marinum* strains cultured in nutrient-rich broth (7H9-OADC) at 33ºC. **b** Growth curve of wild type versus Δ*3581* mutant bacteria during infection of primary mouse macrophage (BMDM) cells. Data is mean ± SD; n = 3 independent replicates. Statistical analysis used an unpaired two-tailed t test; *$p<0.05$. Source data are provided as a Source Data file.

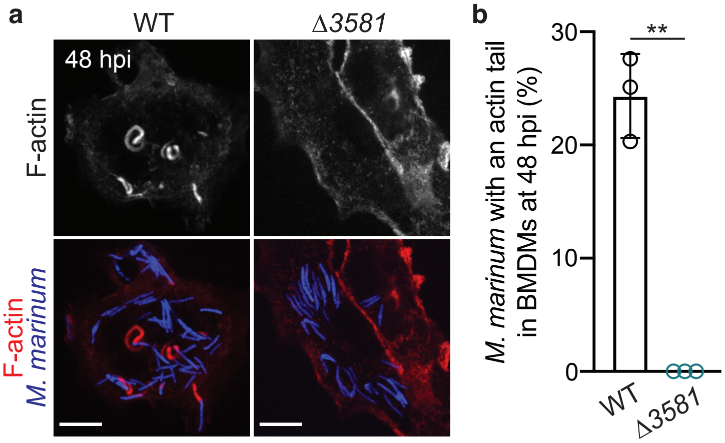

a
WT
Δ3581
48 hpi
F-actin
F-actin
M. marinum
b
M. marinum with an actin tail in BMDMs at 48 hpi (%)
30
20
10
0
**
WT
Δ3581

Supplementary Fig. 3. Δ*MMAR_3581* bacteria are unable to stimulate actin-based motility during infection of hematopoietic primary mouse macrophage cells. a Representative micrograph of wild type or Δ*3581 M. marinum* (blue; EBFP2) associated with F-actin (red; Alexa 561 phalloidin) in BMDM host cells at 48 hpi. Scale bar is 5 µm. **b** Percentage of actin tails for either wild type or Δ*3581* bacteria at 48 hpi of infected BMDM host cells. Data is mean ± SD; n = 3 biological replicates. A paired two-tailed t test was used; **p<0.01. Source data are provided as a Source Data file.

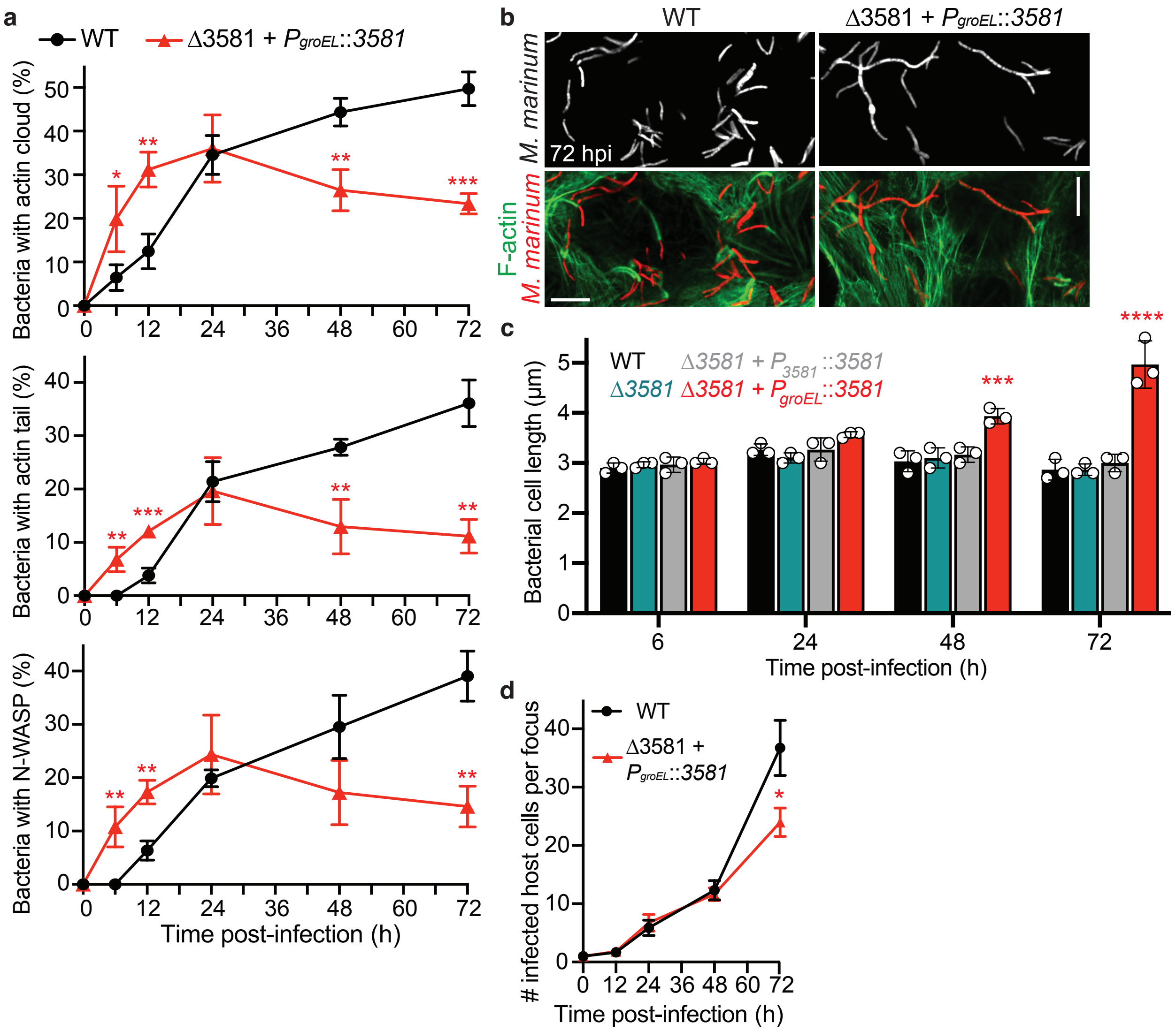

a
WT
Δ3581 + PgroEL::3581
Bacteria with actin cloud (%)
Bacteria with actin tail (%)
Bacteria with N-WASP (%)
Time post-infection (h)
b
WT
Δ3581 + PgroEL::3581
M. marinum
72 hpi
F-actin
M. marinum
c
WT
Δ3581
Δ3581 + P3581::3581
Δ3581 + PgroEL::3581
Bacterial cell length (μm)
Time post-infection (h)
d
WT
Δ3581 + PgroEL::3581
infected host cells per focus
Time post-infection (h)

Supplementary Fig. 4. *MMAR_3581* overexpression results in earlier actin-based motility, but also causes bacterial filamentation during infection. a Time course graphs of wild type or MMAR_3581 overexpressing *M. marinum* colocalized with either F-actin clouds, F-actin tails, or N-WASP. **b** Representative micrographs of either wild type or *MMAR_3581* overexpressing *M. marinum* during infection of U2OS cells at 72 hpi. Scale bar is 5 μm. **c** Cell length measurements of *M. marinum* wild type, Δ*3581*, Δ*3581* + P_{3581}-*3581*, Δ*3581* + P_{groEL}-*3581* over the course of an infection of U2OS cells. **d** Time course graph indicating the number of host U2OS cells per infectious focus during infection of wild type or Δ*3581* + P_{groEL}-*3581 M. marinum*. Data in **a**, **c**, and **d** are the mean ± SD; n = 3 biological replicates. Statistical analyses in **a** and **d** used a unpaired two-tailed t test and **c** used a one-way ANOVA with post-hoc Tukey test was used where each group was compared to wild type; *$p<0.05$, **$p<0.01$, ***$p<0.001$, and ****$p<0.0001$. Source data are provided as a Source Data file.

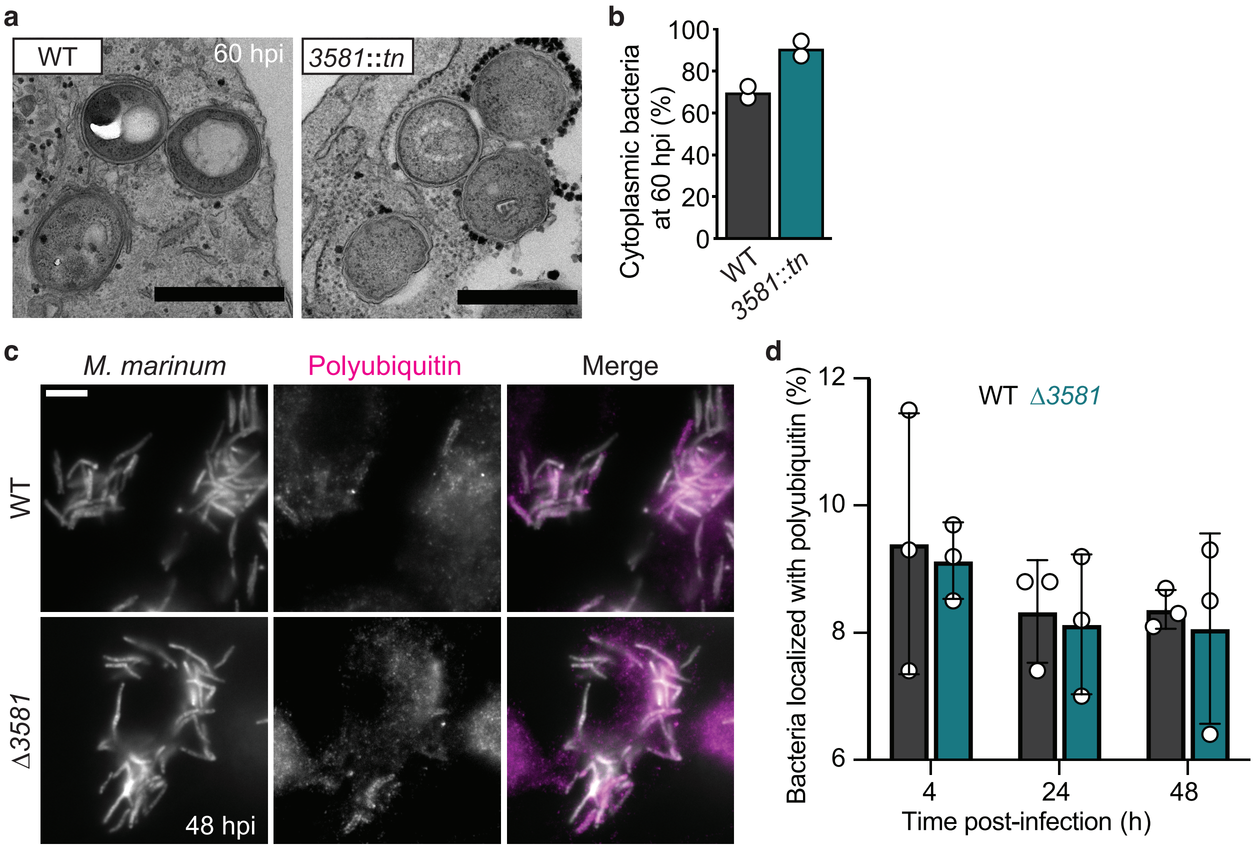
a
WT
60 hpi
3581::tn
b
Cytoplasmic bacteria at 60 hpi (%)
100
80
60
40
20
0
WT
3581::tn
c
M. marinum
Polyubiquitin
Merge
WT
Δ3581
48 hpi
d
Bacteria localized with polyubiquitin (%)
12
10
8
6
WT Δ3581
4
24
48
Time post-infection (h)

Supplementary Fig. 5. *MMAR_3581* deficient bacteria escape from the mycobacterial containing vacuole into the host cytosol. a Representative transmission electron micrographs of host U2OS cells infected with either wild type or *3581*::*tn* bacteria to assess presence of a vacuolar membrane at 60 hpi. Scale bar is 0.5 µm. **b** Quantification of cytoplasmic wild type or *3581*::*tn M. marinum* during infection of U2OS cells at 60 hpi. **c** Representative micrograph of either wild type or Δ*3581 M. marinum* (gray; EBFP2) colocalization with polyubiquitin (magenta; anti-polyubiquitin) during infection of BMDM host cells at 48 hpi. Scale bar is 3 µm. **d** Time course analysis of the frequency of either wild type or Δ*3581* bacteria colocalized with polyubiquitin. Data is the mean ± SD; **b** is n = 2 biological replicates, **d** is n = 3 biological replicates. An unpaired t test was used. Source data are provided as a Source Data file.

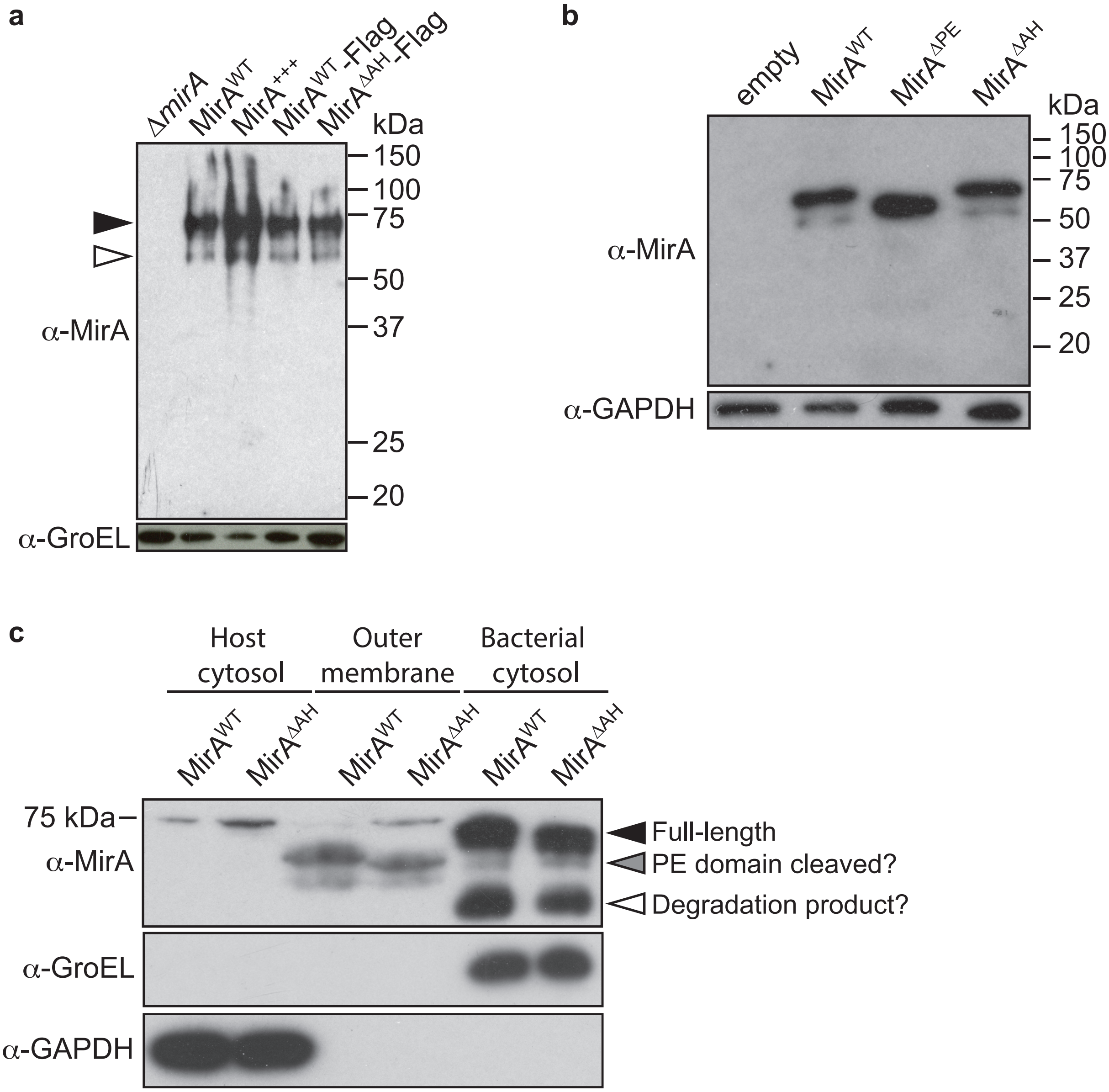

a
ΔmirA
MirA^{WT}
MirA^{+++}
MirA^{WT}-Flag
MirA$^{\Delta AH}$-Flag
kDa
150
100
75
50
37
25
20
α-MirA
α-GroEL
b
empty
MirA^{WT}
MirA$^{\Delta PE}$
MirA$^{\Delta AH}$
kDa
150
100
75
50
37
25
20
α-MirA
α-GAPDH
c
Host cytosol
Outer membrane
Bacterial cytosol
MirA^{WT}
MirA$^{\Delta AH}$
MirA^{WT}
MirA$^{\Delta AH}$
MirA^{WT}
MirA$^{\Delta AH}$
75 kDa
α-MirA
Full-length
PE domain cleaved?
Degradation product?
α-GroEL
α-GAPDH

Supplementary Fig. 6. MirA variant expression levels and subcellular localization. **a** Expression of MirA variants from whole bacterial cell lysates during infection of U2OS cells at 48 hpi. Closed arrow represents full-length MirA, while the open arrowhead likely represents the PE domain cleaved form. GroEL2 expression is used as the loading control. **b** Ectopically expression of MirA variants in U2OS cells. GAPDH is used as the loading control. **c** Subcellular fractionation probing for MirA or MirA$^{\Delta AH}$ in the host cytosol, genapol supernatant, or genapol-extracted bacterial pellets. GroEL2 is used as a control for the bacterial cytosol and GAPDH is a control for the host cytosol. Data shown in **a**-**c** are representative of at least three independent experiments. Source data are provided as a Source Data file.

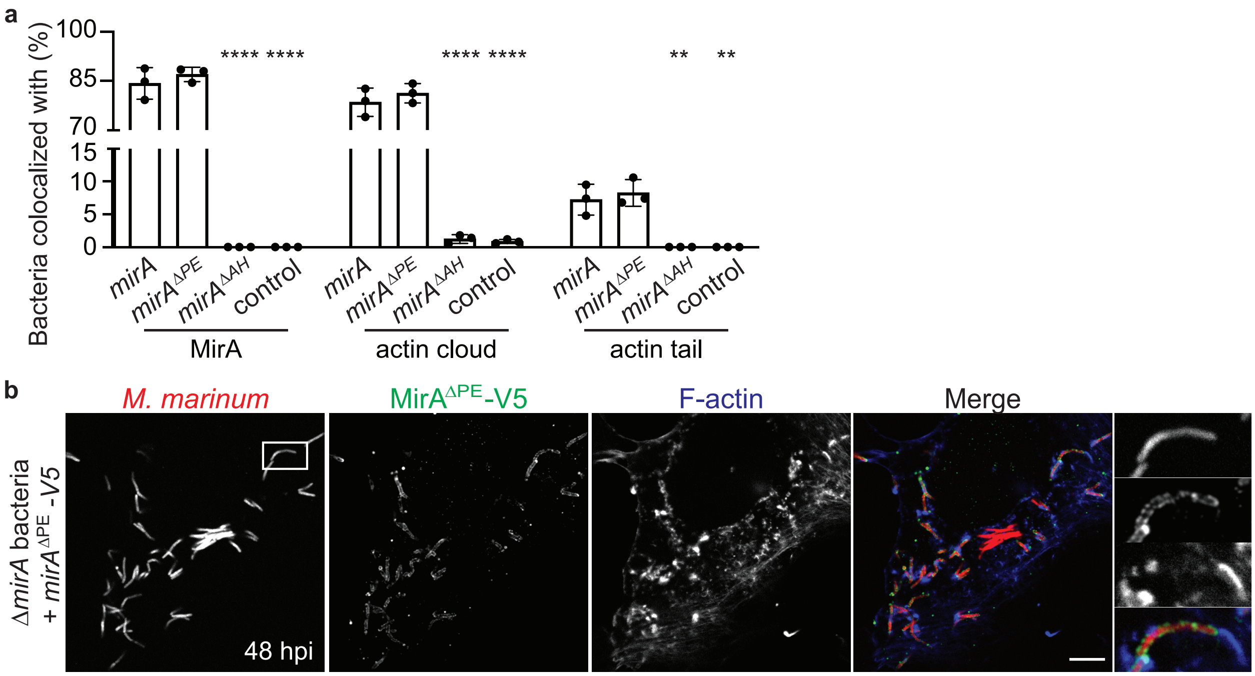
a
Bacteria colocalized with (%)
100
85
70
15
10
5
0
**** ****
**** ****
** **
mirA
mirAΔPE
mirAΔAH
control
MirA
actin cloud
actin tail
b
M. marinum
MirAΔPE-V5
F-actin
Merge
ΔmirA bacteria
+ mirAΔPE -V5
48 hpi

Supplementary Fig. 7. Colocalization measurements for *mirA* complementation *in trans*. a Percent of Δ*mirA* bacteria colocalized with either MirA, an actin cloud, or an actin comet tail in host cells ectopically expressing either MirA, $MirA^{\Delta PE}$, $MirA^{\Delta AH}$, or an empty vector control. Data is mean ± SD; n = 3 biological replicates. A one-way ANOVA with post-hoc Tukey test was used in where each group was compared to wild type; **p<0.01 and ****p<0.0001. **b** Ectopically expressed $MirA^{\Delta PE}$-V5 localizes to the surface of Δ*mirA* bacteria to stimulate actin polymerization at 48 hpi. White box indicates zoom panel, scale bar is 3 μm, and images are representative of at least three independent experiments. Source data are provided as a Source Data file.

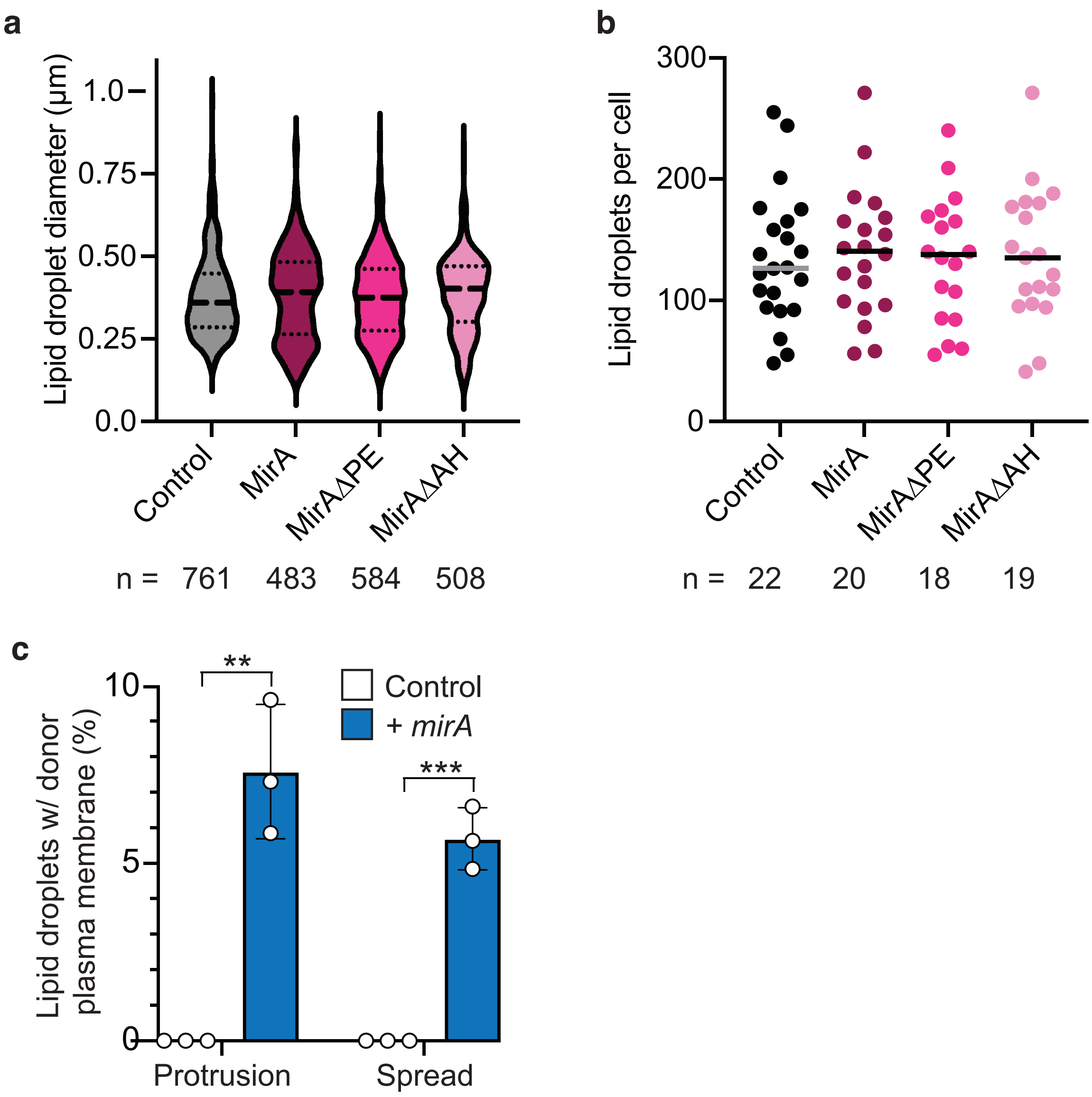
a
Lipid droplet diameter (µm)
1.0
0.75
0.50
0.25
0.0
Control
MirA
MirAΔPE
MirAΔAH
n = 761 483 584 508
b
Lipid droplets per cell
300
200
100
0
Control
MirA
MirAΔPE
MirAΔAH
n = 22 20 18 19
c
Lipid droplets w/ donor plasma membrane (%)
10
5
0
**
***
Control
+ *mirA*
Protrusion
Spread

Supplementary Fig. 8. Host lipid droplet number and size is not impacted by ectopic MirA expression. **a** Measurements of host lipid droplet diameter in A549 cells ectopically expressing MirA, MirA$^{\Delta PE}$, or MirA$^{\Delta AH}$. **b** Number of lipid droplets per A549 cell ectopically expressing MirA, MirA$^{\Delta PE}$, or MirA$^{\Delta AH}$. For **a**, Violin plots outline the 25th and 75th percentile, midline denotes median. For **b**, the bar indicates mean value. For **a** and **b**, then is shown below the figure for both and is the aggregate of three independent experiments. A one-way ANOVA on ranks was used in **a** and one-way ANOVA posthoc Tukey was used **b** where each group was compared to wild type. **c** Quantification of MirA-coated lipid droplets associated with the RFP-CAAX labeled plasma membrane from a donor cell in either a protrusion or vacuole into a recipient cell, related to Fig. 3d. Data is the mean ± SD; n = 3 biological replicates. An unpaired two-tailed t test was used; **p<0.01 and ***p<0.001. Source data are provided as a Source Data file.

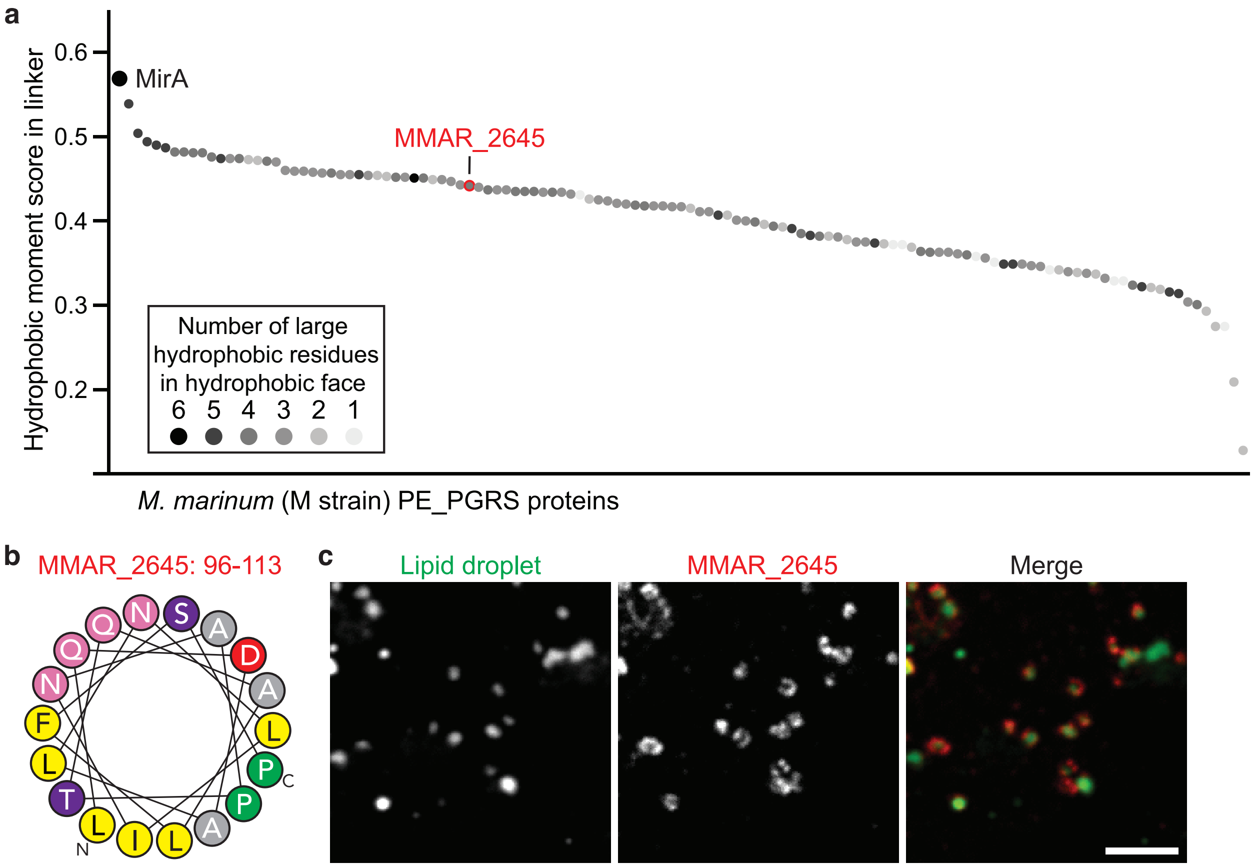
a
Hydrophobic moment score in linker
0.6
0.5
0.4
0.3
0.2
MirA
MMAR_2645
Number of large hydrophobic residues in hydrophobic face
6 5 4 3 2 1
M. marinum (M strain) PE_PGRS proteins
b
MMAR_2645: 96-113
N S A D A L P P A L I L T L F N Q Q
N
C
c
Lipid droplet
MMAR_2645
Merge

Supplementary Fig. 9. Detection of candidate amphipathic helices within *M. marinum* PE_PGRS proteins. a *M. marinum* PE_PGRS proteins were assessed for a putative amphipathic helix in the linker region between their PE and PGRS domains. The best hydrophobic moment score is displayed for each PE_PGRS protein. Additionally, the number of large hydrophobic residues (I, F, L, M, W, Y) within the hydrophobic face, a predictive factor of amphipathic helix insertion into phospholipid monolayers, is shown in greyscale (*inset*). Further details are provided in Supplementary Data 1a. **b** A candidate amphipathic helix in MMAR_2645, residues 96-114. **c** Full-length MMAR_2645-V5, encoding a more representative amphipathic helix hydrophobic moment score, was ectopically expressed in U2OS cells and assessed for localization at the surface of lipid droplet organelles. Scale bar is 2 μm and images are representative of three independent experiments.

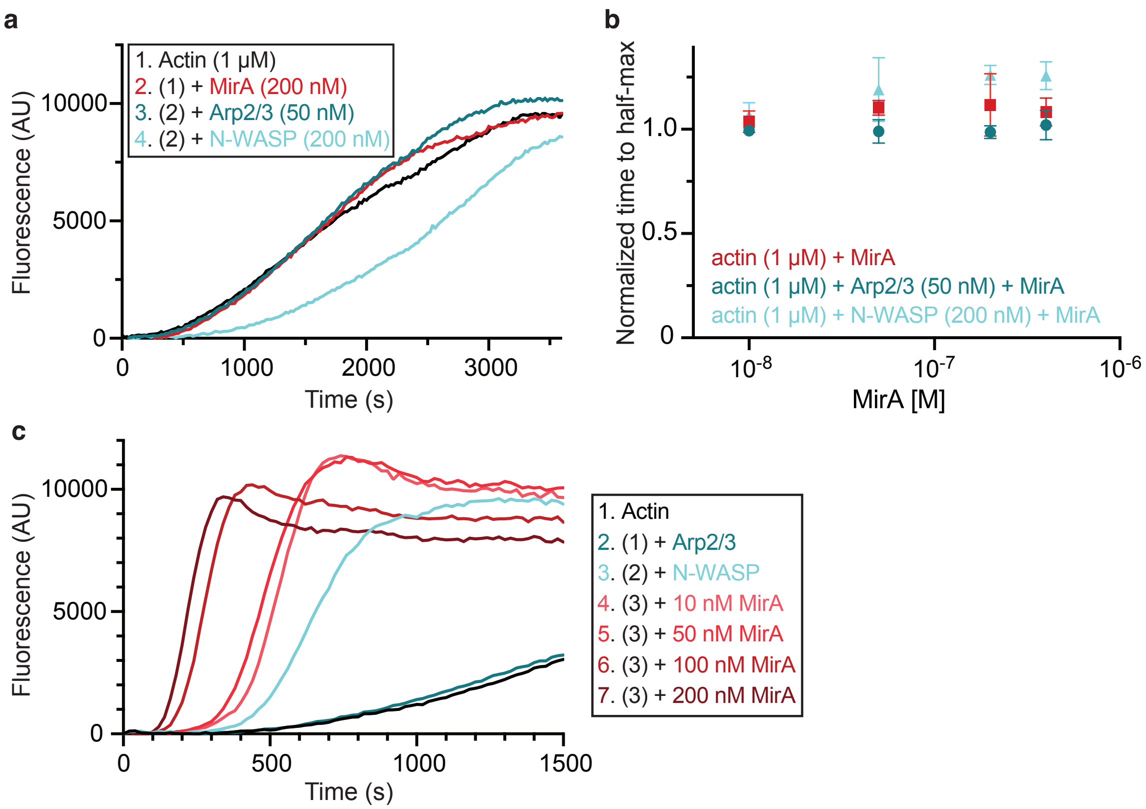
a
1. Actin (1 μM)
2. (1) + MirA (200 nM)
3. (2) + Arp2/3 (50 nM)
4. (2) + N-WASP (200 nM)
Fluorescence (AU)
Time (s)
b
Normalized time to half-max
actin (1 μM) + MirA
actin (1 μM) + Arp2/3 (50 nM) + MirA
actin (1 μM) + N-WASP (200 nM) + MirA
MirA [M]
c
1. Actin
2. (1) + Arp2/3
3. (2) + N-WASP
4. (3) + 10 nM MirA
5. (3) + 50 nM MirA
6. (3) + 100 nM MirA
7. (3) + 200 nM MirA
Fluorescence (AU)
Time (s)

Supplementary Fig. 10. MirA is a direct N-WASP activating protein. a An additional pyrene actin (1 μM, 10% labeled) polymerization reaction trace with the addition of either MirA (200 nM), MirA (200 nM) and the Arp2/3 complex (50 nM), or MirA (200 nM) and N-WASP (200 nM), related to Fig. 4f. **b** The time to half-maximum fluorescence of the pyrene-labeled actin polymerization curves with MirA (200 nM), the Arp2/3 complex (50 nM) and MirA, or N-WASP (200 nM) and MirA, which has been normalized to actin alone. Data is mean ± SD; n = 3 technical replicates. **c** An additional example of pyrene-labeled actin (1 μM, 10% labeled) polymerization reactions with the Arp2/3 complex (50 nM), N-WASP (200 nM), and increasing MirA concentrations, related to Fig. 4g. Source data are provided as a Source Data file.

Fig. 3a

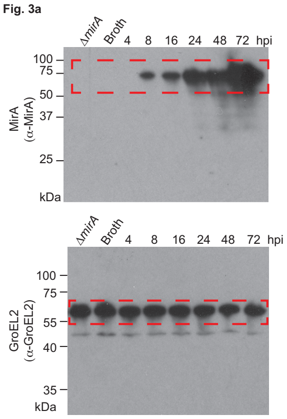

Fig. 7c

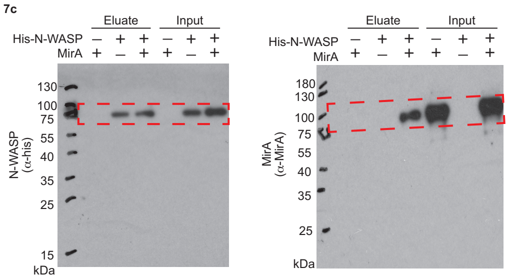

Fig. 6b

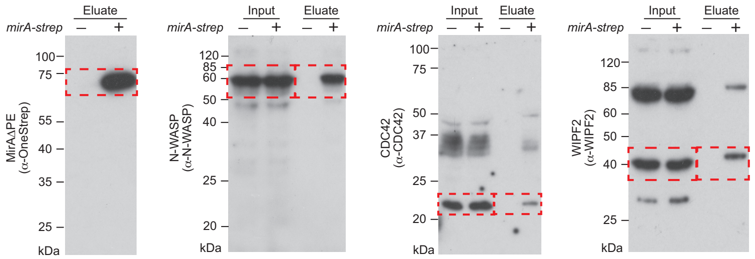

Fig. 6c

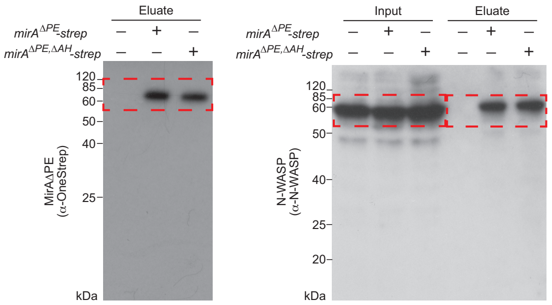

Fig. 6D

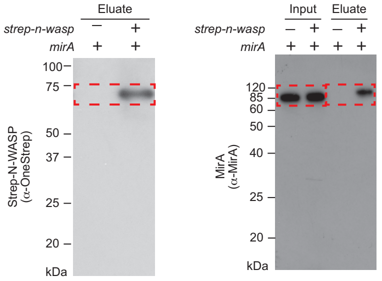

Supplementary Fig. 6b

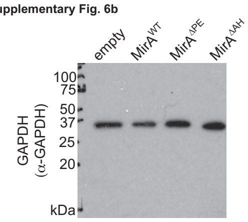

Supplementary Fig. 6c

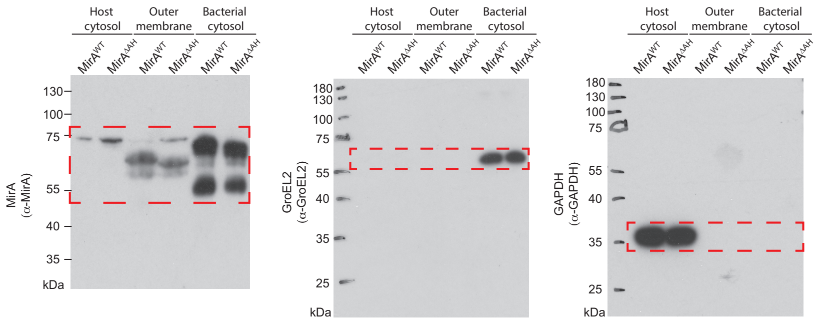

Supplementary Fig. 11. Uncropped immunoblots displayed in the study.
