# Supplementary material for: A glycine-rich PE_PGRS protein governs mycobacterial actin-based motility
Source: Nat Commun. 2022 Jun 24;13:3608. doi: 10.1038/s41467-022-31333-0 (PMC9232537; doi:10.1038/s41467-022-31333-0)
Supplement: Supplementary file 2 — Reporting Summary [file 41467_2022_31333_MOESM2_ESM.pdf]

nature portfolio

Corresponding author(s): Matthew D. Welch & Norbert S. Hill

Last updated by author(s): 6/8/2022

Reporting Summary

Nature Portfolio wishes to improve the reproducibility of the work that we publish. This form provides structure for consistency and transparency in reporting. For further information on Nature Portfolio policies, see our Editorial Policies and the Editorial Policy Checklist.

Statistics

For all statistical analyses, confirm that the following items are present in the figure legend, table legend, main text, or Methods section.

n/a	Confirmed	
☐	☒	The exact sample size (*n*) for each experimental group/condition, given as a discrete number and unit of measurement
☐	☒	A statement on whether measurements were taken from distinct samples or whether the same sample was measured repeatedly
☐	☒	The statistical test(s) used AND whether they are one- or two-sided<br>*Only common tests should be described solely by name; describe more complex techniques in the Methods section.*
☒	☐	A description of all covariates tested
☒	☐	A description of any assumptions or corrections, such as tests of normality and adjustment for multiple comparisons
☐	☒	A full description of the statistical parameters including central tendency (e.g. means) or other basic estimates (e.g. regression coefficient) AND variation (e.g. standard deviation) or associated estimates of uncertainty (e.g. confidence intervals)
☐	☒	For null hypothesis testing, the test statistic (e.g. *F*, *t*, *r*) with confidence intervals, effect sizes, degrees of freedom and *P* value noted<br>*Give P values as exact values whenever suitable.*
☒	☐	For Bayesian analysis, information on the choice of priors and Markov chain Monte Carlo settings
☒	☐	For hierarchical and complex designs, identification of the appropriate level for tests and full reporting of outcomes
☒	☐	Estimates of effect sizes (e.g. Cohen's *d*, Pearson's *r*), indicating how they were calculated

Our web collection on statistics for biologists contains articles on many of the points above.

Software and code

Policy information about availability of computer code

Data collection	Metamorph (v. 7.8), MassLynx software (v. 4.1), Tecan with Magellan (v.7)
Data analysis	FIJI (v. 2), PRISM (v. 8 and v. 9), Adobe Illustrator (v. 25.4.1), Microsoft Excel (v. 16.53), HELIQUEST (v. 2), MiST (v. main.e2da2b0)

For manuscripts utilizing custom algorithms or software that are central to the research but not yet described in published literature, software must be made available to editors and reviewers. We strongly encourage code deposition in a community repository (e.g. GitHub). See the Nature Portfolio guidelines for submitting code & software for further information.

Data

Policy information about availability of data

All manuscripts must include a data availability statement. This statement should provide the following information, where applicable:

- Accession codes, unique identifiers, or web links for publicly available datasets
- A description of any restrictions on data availability
- For clinical datasets or third party data, please ensure that the statement adheres to our policy

Genomic locations of transposon insertion were determined using BLAST against GenBank/NCBI accession NZ_HG917972. Data acquired by mass spectrometry (Fig. 6a) were searched against a database containing SWISS-PROT Human protein sequences (accessed 28 August 2018) and examined by the STRING database (accessed 10 May 2020). All other data that support the findings of this study and a detailed description of the methods used are available in the manuscript, Supplementary Information, or source data.

Field-specific reporting

Please select the one below that is the best fit for your research. If you are not sure, read the appropriate sections before making your selection.

☒ Life sciences ☐ Behavioural & social sciences ☐ Ecological, evolutionary & environmental sciences

For a reference copy of the document with all sections, see nature.com/documents/nr-reporting-summary-flat.pdf

Life sciences study design

All studies must disclose on these points even when the disclosure is negative.

Sample size	No statistical methods were used to predetermine sample size. We used numbers that are standard practice in the field that were compiled from three or more independent replicates. An n = 2 was used in two cases: (1) the TEM analysis probing for cytosolic/ vacuolar bacteria (Supplementary Fig. 5b) and (2) the AP-MS experiment (Fig. 6a; Supplementary Data 2).
Data exclusions	There were no data exclusion in this study.
Replication	The number of replicates for a particular experiment is clearly denoted in either the Result or Methods sections, figure, figure legend, or source data. Experiments are at least three independent biological or technical replicates (with the exception of Supplementary Fig. 5b and Fig. 6a where the n = 2) that were executed weeks or months apart. Key phenotypes are consistent across different bacterial strains (e.g. mirA::tn vs. #mirA), cell lines (U2OS vs. macrophage), and media and all attempts to replicate data were successful.
Randomization	Consistent with practices in the field, allocation of samples into experimental groups was not random.
Blinding	Blinding was employed in acquisition and analysis for the following situations. Positive and negative plate controls in the transposon insertion screen that identified bacteria defective in cell-to-cell spread. TEM analysis probing for cytosolic/vacuolar bacteria (Supplementary Fig. 5a and b). Probing for N-WASP and Arp2/3 on the surface of lipid droplets ± mirA expression (Fig. 4d and e). Velocity of BSA- or MirA-coated polystyrene beads (Fig. 7b). Blinding was not feasible for imaging experiments comparing WT to the ΔmirA bacteria because of an obvious visual difference between bacterial distribution. Otherwise, consistent with practices in the field, investigators were not blinded in acquisition or analysis. Additionally, all experiments were replicated and well-controlled through inclusion of wild-type, mutant, and complemented strains.

Reporting for specific materials, systems and methods

We require information from authors about some types of materials, experimental systems and methods used in many studies. Here, indicate whether each material, system or method listed is relevant to your study. If you are not sure if a list item applies to your research, read the appropriate section before selecting a response.

Materials & experimental systems

n/a	Involved in the study	
☐	☒	Antibodies
☐	☒	Eukaryotic cell lines
☒	☐	Palaeontology and archaeology
☒	☐	Animals and other organisms
☒	☐	Human research participants
☒	☐	Clinical data
☒	☐	Dual use research of concern

Methods

n/a	Involved in the study	
☒	☐	ChIP-seq
☒	☐	Flow cytometry
☒	☐	MRI-based neuroimaging

Antibodies

Antibodies used	Commercial antibodies: rabbit anti-CDC42 (GeneTex; Cat# GTX100904; RRID:AB_1240608), rabbit anti-WIPF2 (Sigma-Aldrich; Cat# HPA024467; RRID:AB_1858842), mouse anti-V5 (Thermo Fisher Scientific; Cat# R960-25; RRID:AB_2556564), rabbit anti-FLAG (Cell Signaling; Cat# 2368S; RRID: AB_10694612), mouse anti-GAPDH (Ambion; Cat# AM4300; RRID: AB_437392), mouse anti-GroEL2 from M. tuberculosis (BEI Resources, NIAID, NIH; NR-19363), mouse anti-polyubiquitin (Enzo Life Sciences; Cat# BML-PW8805-0500; RRID: AB_2052280), mouse anti-His (GenScript; Cat# A00186, RRID:AB_914704).<br><br>Non-commercial antibodies: Guinea pig anti-N-WASP (reference is PMID: 20175130, provided by UC Berkeley/Matthew Welch), Rabbit anti-P34 (reference is PMID: 9000076, provided by UC Berkeley/Matthew Welch), and Rabbit anti-MirA (this work).<br><br>Secondary antibodies: Conjugated to Alexa fluorophores (Invitrogen; A21131 (goat anti-mouse 488), A11008 (goat anti-rabbit 488), A11073 (goat anti-guinea pig 488), A11075 (goat anti-guinea pig 568), A11036 (goat anti-rabbit 568), A11004 (goat anti-mouse 568)) or conjugated to HRP (Santa Cruz Biotechnology, sc-2005 (goat anti-mouse), sc-2357 (mouse anti-rabbit), sc-516102 (mouse IgG kappa); abcam, 6908 (goat anti-guinea pig); Pierce PA1-26848 (anti-avidin).
Validation	Commercial antibodies were selected using guidance of the "antibodypedia" website (https://www.antibodypedia.com/) detailing antibody validation. Anti-CDC42 has been validated for immunoblotting in human and mouse cells by the manufacturer (GeneTex). Anti-WIPF2 as been validated for immunoblotting in human and mouse cells by the manufacturer (Sigma-Aldrich). Anti-V5 has been validated for immunoblotting and immunofluorescence in human and mouse cells by the manufacturer (Thermo Fisher Scientific). Anti-FLAG has been validated for immunoblotting and immunofluorescence in human and mouse cells by the manufacturer (Cell Signaling). Anti-GAPDH has been validated in human and mouse cells for immunoblotting by the manufacturer (Ambion). Anti-GroEL2 has been validated for immunoblotting by the manufacturer in human and mouse cells (BEI Resources, NIAID, NIH). Anti-polyubiquitin has been validated for immunofluoresence in human and mouse cells by the manufacturer (Enzo Life Sciences). And, anti-His has been validated for immunoblotting in human and mouse cells by the manufacturer (GenScript).<br><br>Non-commercial antibodies have previously been vetted or by this study. Anti-N-WASP, derived against the WCA domain, has been previous described and validated for immunoblotting and immunofluorescence by the study that generated this antibody (PMID: 20175130). Anti-P34 was validated for immunoblotting and immunofluorescence by the study that generated this antibody (PMID: 9230079). Anti-MirA was raised and affinity purified as described in Methods section, and this study demonstrates it to be specific for MirA by immunoblotting (Supplementary Fig. 6 and 11).

Eukaryotic cell lines

Policy information about cell lines

Cell line source(s)	U2OS (RRID:CVCL_0042), Raw 264.7 (RRID:CVCL_0493), HEK293 (RRID:CVCL_0045), Sf9 (RRID:CVCL_0549) and A549 (RRID:CVCL_0023) were obtained from the University of California, Berkeley Cell Culture Facility (UCB-CCF). BMDMs were generated from the femurs of female C57BL/6 mice (Charles River Laboratories, Wilmington, MA).
Authentication	Cell lines acquired from the University of California, Berkeley Cell Culture Facility (UCB-CCF) had been authenticated using short-tandem-repeat analysis.
Mycoplasma contamination	All cell lines were confirmed to be mycoplasma-negative by DAPI staining and fluorescence microscopy screening at the UC Berkeley Cell Culture Facility.
Commonly misidentified lines<br>(See ICLAC register)	No commonly misidentified cell lines were used in this study.
