# Supplementary material for: A glycine-rich PE_PGRS protein governs mycobacterial actin-based motility
Source: Nat Commun. 2022 Jun 24;13:3608. doi: 10.1038/s41467-022-31333-0 (PMC9232537; doi:10.1038/s41467-022-31333-0)
Supplement: Supplementary file 4 — Description of Additional Supplementary Files [file 41467_2022_31333_MOESM4_ESM.pdf]

Description of Additional Supplementary Files

File Name: Supplementary Movie 1
Description: **MirA-transfected cells exhibit actin rocketing lipid droplets in time lapse microscopy,** related to Fig. 3. U2OS cells stably expressing F-tractin-mCherry (a fluorescent F-actin marker) were transfected with a plasmid expressing *mirA* (pBH261). Images were captured from a single plane time-lapse every 5 s for 10 min. The video is displayed at 8 frames/s and is repeated twice, then played with object tracking. Timestamp shows min: sec and scale bar is 3 µm.

File Name: Supplementary Movie 2
Description: **Lipid droplets actin rocket in MirA-transfected cells,** related to Fig. 3b. This movie is a cropped portion of Supplementary Movie 1 (from 330 s to 580 s) and corresponds to snapshots in Fig. 3b. Images were captured from a single plane time-lapse every 5 s for 250 s displayed at 8 frames/s and is then repeated with object tracking. Timestamp shows min: sec and scale bar is 1 µm.

File Name: Supplementary Movie 3
Description: **MirA-transfected cells with labeled lipid droplets.** U2OS cells stably expressing F-tractin-mCherry (red; a fluorescent F-actin marker) were transfected with a plasmid expressing *mirA*$^{\Delta PE}$ (pBH310) and stained for lipid droplets (green; Bodipy 493/503). F-tractin-mCherry alone is shown below. Images were captured every 5 s for 135 s displayed at 6 frames/s and the video is repeated thrice. Timestamp shows min: sec and scale bar is 5 µm.

File Name: Supplementary Movie 4
Description: **Purified MirA coated onto polystyrene beads generate actin tails in a cell-free extract,** related to Fig. 4c. Polymerization of rhodamine-actin (red) on 1 µm fluorescent polystyrene beads (green) coated with purified MirA in *Xenopus* extract. Images were captured every 5 s for 2.25 min displayed at 6 frames/s and video is repeated 5 times. Timestamp shows min: sec and scale bar is 5 µm.

File Name: Supplementary Movie 5
Description: **MirA-coated polystyrene beads exhibit sustained motility in cell-free extract,** related to Fig. 4d. 0.5 µm fluorescent polystyrene beads coated with either BSA (left) or purified MirA (right) in *Xenopus* extract. Images were captured every 2.5 s for 175 s displayed at 8 frames/s. The movie is played once, then repeated with object tracking. Timestamp shows time in seconds and scale bar is 30 µm.

File Name: Supplementary Data 1
Description: **Putative amphipathic helices in *M. marinum* and *M. tuberculosis* PE_PGRS proteins,** related to Supplementary Fig. 9b, c. Bioinformatically assessing the linker regions between the PE and PGRS domains of either (A) *M. marinum* (M strain) or (B) *M. tuberculosis* (H37Rv) PE_PGRS proteins for amphipathic helices. PE_PGRS proteins are ranked by hydrophobic moment score and the number of large, non-polar residues within the hydrophobic face is listed.

File Name: Supplementary Data 2
Description: **MirA-Host protein-protein interaction dataset,** related to Fig. 4b. AP-MS dataset from two independent experiments (A and B). Host proteins identified from the AP-MS with a NSAF differential (MirA-control) value > 0.0002 are listed (full dataset available upon request). (C) MiST scores of proteins scoring ≥ 0.75.

File Name: Supplementary Data 3
Description: **Plasmids, *M. marinum* strains, and antibodies used in this work,** related to Methods.
